# Sequence duplication in 3′ UTR modulates virus replication and virulence of Japanese encephalitis virus

**DOI:** 10.1080/22221751.2021.2016354

**Published:** 2021-12-21

**Authors:** Qiu-Yan Zhang, Si-Qing Liu, Xiao-Dan Li, Jia-Qi Li, Ya-Nan Zhang, Cheng-Lin Deng, Hong-Lei Zhang, Xu-Fang Li, Chun-Xiao Fang, Feng-Xia Yang, Bo Zhang, Yi Xu, Han-Qing Ye

**Affiliations:** a The Joint Center of Translational Precision Medicine, Department of Infectious Diseases, Guangzhou Institute of Pediatrics, Guangzhou Women and Children's Medical Center, Guangzhou, People’s Republic of China; b The Joint Center of Translational Precision Medicine, Wuhan Institute of Virology, Chinese Academy of Sciences, Wuhan, People’s Republic of China; c Key Laboratory of Special Pathogens and Biosafety, Wuhan Institute of Virology, Center for Biosafety Mega-Science, Chinese Academy of Sciences, Wuhan, People’s Republic of China; d School of Medicine, Hunan Normal University, Changsha, People’s Republic of China; e University of Chinese Academy of Sciences, Beijing, People’s Republic of China; f College of Veterinary Medicine, Henan Agricultural University, Zhengzhou, People’s Republic of China

**Keywords:** Japanese encephalitis virus, 3′ UTR, virus replication, DB duplication, viral virulence

## Abstract

Japanese encephalitis virus (JEV), an important neurotropic pathogen, belongs to the genus *Flavivirus* of the family *Flaviviridae* and has caused huge threat to public health. It is still obscure regarding the functions of stem-loop (SL) and dumbbell (DB) domains of JEV 3′ UTR in viral replication and virulence. In the current study, using the infectious clone of JEV SA14 strain as a backbone, we constructed a series of deletion mutants of 3′ UTR to investigate their effects on virus replication. The results showed that partial deletions within SL or DB domain had no apparent effects on virus replication in both mammalian (BHK-21) and mosquito (C6/36) cells, suggesting that they were not involved in viral host-specific replication. However, the entire SL domain deletion (ΔVR) significantly reduced virus replication in both cell lines, indicating the important role of the complete SL domain in virus replication. The revertant of ΔVR mutant virus was obtained by serial passage in BHK-21 cells that acquired a duplication of DB domain (DB-dup) in the 3′ UTR, which greatly restored virus replication as well as the capability to produce the subgenomic flavivirus RNAs (sfRNAs). Interestingly, the DB-dup mutant virus was highly attenuated in C57BL/6 mice despite replicating similar to WT JEV. These findings demonstrate the significant roles of the duplicated structures in 3′ UTR in JEV replication and provide a novel strategy for the design of live-attenuated vaccines.

## Introduction

Flaviviruses are a large group of arborviruses within the *Flaviviridae* family, including many important human pathogens, such as Japanese encephalitis virus (JEV), dengue virus (DENV), West Nile virus (WNV), yellow fever virus (YFV), Zika virus (ZIKV) and tick-borne encephalitis virus (TBEV). Among them, JEV is the most prevalent cause of viral encephalitis. Since its first outbreak in Japan in 1924 [1], JEV has become epidemic in most countries in Asia and Oceania [2,3]. Recently, autochthonous cases of JEV in humans or birds have been reported in Africa and Europe [4], suggesting the widespread of JEV in non-endemic regions. Although SA14-14-2, a live-attenuated JEV vaccine, has been widely used in China and other Asia countries, the safety concern remains a major hurdle for its acceptance and application around the world. So far, there is no specific antiviral therapy available for JEV infection. It is important to investigate the mechanism of virus replication for antivirals and vaccines development.

JEV genome is a single-stranded, positive-sense RNA, approximately 11 kb in length, which contains a large open reading frame (ORF) flanked by 5′ untranslated region (5′ UTR) and 3′ UTR. The ORF encodes a polyprotein which is cleaved by viral or cellular proteases into three structural proteins (capsid, C; premembrane, prM; envelope, E) and seven nonstructural proteins (NS1, NS2A, NS2B, NS3, NS4A, NS4B and NS5) for viral particle formation and genome replication, respectively. The UTRs contain various RNA structures which are involved in virus replication [5,6], host-adaptation [7–9] and pathogenicity [10,11], and are new targets for antivirals and vaccines design in recent years.

**Figure 1.**
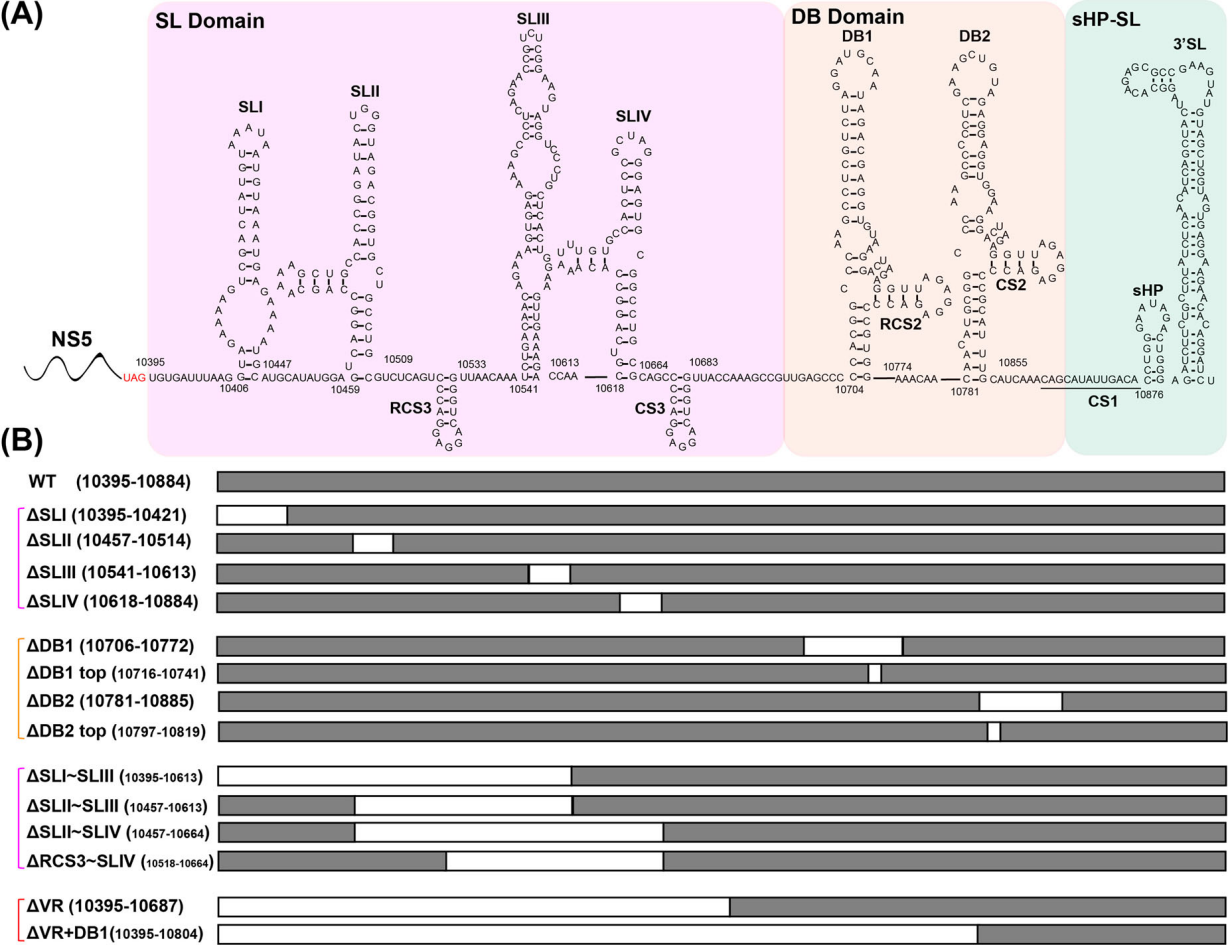
Schematic of JEV 3′ UTR and deletion mutants. (A) Secondary structure diagram of JEV SA14 full-length 3′ UTR. Three domains are divided into purple, orange or green boxes, respectively. Stem-loop (SL), dumbbell (DB) structures, conserved sequences (CS) or repeated CS (RCS) were shown in order. (B) Systematic presentation of deletion mutants in SL and DB domains which are divided into four groups: individual structure deletion in domain SL, individual structure deletion in domain DB, combined structures deletion in domain SL, and large fragment deletion in SL and DB domains. Deletion sequences were exhibited in blank frames, and the details of missing locations in the genome were labelled.

The 5′ UTR of JEV consists of two stem-loop structures (SLA and SLB), which are essential elements for NS5 recruitment and viral RNA synthesis [12,13]. Based on the sequence variability and the predicted secondary structures, JEV 3′ UTR is sequentially divided into three domains: the stem-loop (SL) domain, the dumbbell (DB) domain and the 3′-stem-loop (sHP-SL) domain (Figure 1(A)) [14]. The SL domain, which directly follows the NS5 stop codon, is considered as the most highly variable region (VR) in the 3′ UTR and forms four stem-loop (SL) structures (SLI ∼ SLIV). Among them, SLI and SLIII are characteristic of the encephalitic flaviviruses (JEV group) [15], and another two similar structures, SLII and SLIV, are common in mosquito-borne flaviviruses (Figure S1) [16]. The sequences of DB domain are moderately conserved, forming duplicated dumbbell structures (DB1 and DB2). DB1/2 structures are also found in DENV and WNV, while ZIKV and YFV produce an unauthentic DB structure (ΨDB) (Figure S1). The sHP-SL domain is the most conserved region which contains the cyclization sequence (CS1) and distal 3′-stem-loop (3′ SL) structure. For all flaviviruses, CS1 and 3′ SL structures interact with 5′ UTR and play an essential role in genome cyclization and viral RNA synthesis [5,6,17,18], which are indispensable for the virus genome replication. For the less conserved sequences and structures in SL and DB domains, previous studies have demonstrated their various functions during the viral life cycle using different flavivirus models.

Many studies have shown that highly complex RNA elements, SL and DB, are closely related to the efficiency of virus replication [19,20]. Deletions or mutations in these structures greatly affect DENV replication in mammalian cells, but don’t influence virus propagation in mosquito cells [21,22]. It is thus regarded that the duplicated structures may play essential functions in host-specific replication, facilitating viral fitness during host switching and further influencing virus transmission [7,8,22]. Moreover, it was demonstrated that in WNV, DENV, YFV and ZIKV, SL and DB structures are essential elements for the production of subgenomic flavivirus RNAs (sfRNAs) [11,19,23–25]. In addition, deletion or point mutations in SL or DB domain also attenuated viral virulence of DENV, WNV and ZIKV in mice [9,11,26]. The above studies about the functions of the SL and DB domains were mainly focused on DENV, WNV, YFV and ZIKV, whose structures in these two domains are different from JEV. The sequence and structural diversity in domain SL and DB among flaviviruses indicates their specific evolutionary roles in different flaviviruses. To date, extensive structure–function analysis of SL and DB domains in JEV replication is still lacking.

In this study, we investigated the function of SL and DB domains of JEV 3′ UTR by constructing a series of deletion mutants in these two domains. We found that different with DENV, the duplicated structures in JEV SL and DB domains were not involved in host-specific replication as all the mutants showed consistent effects on viral replication in mammalian BHK-21 cells and mosquito C6/36 cells (minimal/no effects caused by most of mutants or a detrimental effect caused by ΔVR mutant). ΔVR mutant harboured a complete deletion of SL domain with very poor replication efficiency in either cell line. Serial passage of ΔVR mutant in BHK-21 cells produced revertant viruses bearing duplicated DB domain sequences (DB-dup) in 3′ UTR, which restored viral replication and sfRNAs production. In addition, we found that the DB-dup mutant virus was attenuated in C57BL/6 mice, although it exhibited similar replication efficiency to wild-type (WT) JEV in cell culture. Overall, these data demonstrate that the structure duplication in 3′ UTR is essential for JEV replication and provide new insights for the design of novel live-attenuated vaccines.

## Materials and methods

### Cell cultures and antibodies

Baby hamster kidney (BHK-21) cells were cultured in Dulbecco’s modified Eagle’s medium (DMEM) containing 10% foetal bovine serum (FBS), 100 units/mL penicillin, and 100 µg/mL streptomycin in 5% CO_2_ at 37 °C. *Aedes albopictus* clone C6/36 (ATCC number: CRL-1660) cells were cultured in RPMI 1640 medium (Gibco) with 10% FBS at 28 °C. The monoclonal antibody 4G2 against the E protein of *flavivirus* was kindly provided by Dr. Qin, Cheng-Feng (Beijing Institute of Microbiology and Epidemiology, China). FITC-conjugated goat anti-mouse IgG was purchased from Protein Tech Group.

### Plasmid construction

The full-length infectious clone of JEV SA14 strain (GenBank accession no. U14163) generated previously in our laboratory was designated as WT and used as the backbone to construct various deletion or revertant mutants [27]. All JEV 3′ UTR deletion mutations were introduced by fusion PCR, and the sequences of the primers used in the construction of the mutant were listed in Table S1. The X*ba*I and X*ho*I restriction sites were used to construct the infectious clones of deletion mutants. For DB-dup mutant construction, the total RNAs extracted from the ΔVR-P12-B infected cells were used as the template of reverse transcription PCR (RT–PCR), and the primer pair JEV-8881-NS1-F and JEV-3’ UTR-HDVr-R was used to amplify fragment A. Fragment B was amplified using JEV SA14 infectious clone as the template using primers JEV-3’ UTR-HDVr-F and PACYC-R. Fusion PCR was performed to obtain large fragments containing DB-dup mutation. The N*de*I and X*ho*I restriction sites were used to construct the DB-dup clone. All constructs were verified by DNA sequencing.

### Production of viruses

The WT and mutant infectious clones were linearized with X*ho*I, followed by *in vitro* transcription using T7 mMESSAGE mMACHINE kit (Thermo Fisher Scientific). About 1 μg WT or mutant RNAs were transfected into BHK-21 and C6/36 cells using DMRIE-C reagent (Invitrogen) following manufacturer’s protocol and cultured at 37°C or 28°C, respectively. The supernatants of WT and mutant RNAs-transfected cells were collected at different time points post transfection.

### Indirect immunity fluorescence (IFA)

At the indicated time points after transfection or infection, the cells were fixed with cold 5% acetone (in methanol) at room temperature for 10 min. After fixation, the cells were incubated with anti-E protein monoclonal antibody 4G2 (1:500, dilution in PBS), followed by incubation with FITC-conjugated goat anti-mouse IgG (Thermo Fisher Scientific, 1:125). Nuclei were stained with DAPI. The images were captured using a Zeiss inverted microscope at ×400 magnification.

### Plaque assay and virus growth kinetics

Virus titre and morphology were detected by the monolayer plaque assay. Briefly, a series of 1:10 dilutions were prepared by diluting 15 μL virus stock with 135 μL DMEM containing 2% FBS, and 100 μL of each dilution was seeded onto 24-well plates containing confluent BHK-21 cells. The infected cells were incubated at 37 °C for 1 h before the medium containing 1% methylcellulose was overlaid. After 3 or 4 days of incubation, the cells were ﬁxed in 3.7% formaldehyde and then stained with 1% crystal violet. The viral titre was calculated as plaque-forming units (PFU)/mL.

The double-layer plaque assay of ΔVR-P12-A/B/C viruses was performed to identify the revertant mutations. The viruses were diluted at 1:10 as described earlier, and after virus incubation for 1 h, the first layer of agar was added. After 3 days of incubation at 37°C with 5% CO_2_, the second layer of agar containing neutral red was added. About 12–24 h later, 12 viral plaque clones were randomly selected to infect BHK-21 cells. Once the infected cells appeared obvious cytopathic effect (CPE), total cellular RNAs were extracted using Trizol reagent (Invitrogen).

For viral growth curve analysis, BHK-21 and C6/36 cells were infected with WT or mutant viruses at a multiplicity of infection (MOI) of 0.1, respectively, and the supernatants were harvested at the indicated time points and stored at −80°C. The viral titres at different time points were determined by the monolayer plaque assay. The growth curves were plotted using Graphpad Prism 8.0 software.

### Generation of recovered viruses

To obtain replication recovered viruses of ΔVR mutant, ΔVR mutant virus was subjected to serial passage in BHK-21 cells. The culture supernatants from the ΔVR mutant RNA-transfected BHK-21 cells were designated as P0 virus. The P0 virus was used for three independent passages (designated as A/B/C strain, respectively). The supernatants were collected and subjected to the next round of passage when the infected cells showed obvious cytopathic effects (CPE). Twelve clones of ΔVR-P12-A/B/C viruses were further purified by the double-layer plaque assay as described above. The total RNAs of each single clone of ΔVR-P12-A/B/C infected cells were extracted and RT–PCR was then carried out using the PrimeScript One Step RT–PCR Kit (Takara) with primers JEV-9841-F and JEV-10977-R (Table S1) to amplify the fragment spanning from NS5 to 3’ UTR. The products were subjected to DNA sequencing to characterize the genetic changes of the viral genome.

### Northern blotting analysis

For JEV genome and sfRNAs detection, total RNAs extracted from BHK-21 cells infected with different mutant viruses were analysed by Northern blotting assay. About 20 μg of RNAs was loaded on a 1.5% agarose-2% formaldehyde gel followed by transfer onto Hybond-N+ membranes (GE healthcare) using capillary migration with 20×SSC buffer (3 M sodium chloride, 0.3 M sodium citrate, pH 7.0). Next, the blots were UV-crosslinked and hybridized with digoxigenin (DIG)-ddUTP-labelled 3′-UTR DNA probes. Membrane blocking, washing and visualization were performed using the DIG High Prime DNA Labelling and Detection Starter Kit I (Roche). The blocking buffer was prepared by diluting the Vial 6 of Kit I (1:10) into maleic acid buffer (0.1 M maleic acid, 0.15 M NaCl, pH 7.5). The composition of the washing buffer was 0.1 M maleic acid, 0.15 M NaCl, pH 7.5 and 0.3% Tween 20 (v/v). The DNA probe used for Northern Blot detection contained the sequence from 10847 to 10977 nt of the viral genome, which was obtained by PCR using the primer pair, JEV-10847-F and JEV-10977-R (Table S1).

### Virulence experiments

For virus virulence experiments, 3-week-old C57BL/6 mice were inoculated intraperitoneally (i.p.) with 1×10^7^ PFU of WT JEV or DB-dup mutant viruses (n = 5), respectively. The survival and body-weight changes of mice were monitored daily for 21 days. Viremia at 1 and 2 days post infection was quantified by monolayer plaque assay in BHK-21 cells as described previously, and the limit of detection was 100 PFU/mL. Mice were dissected, and virus loads in the brain were also detected by plaque assay. The experiments were performed at an animal biosafety level 2 (ABSL-2) facility in Wuhan Institute of Virology under a protocol approved by the Laboratory Animal Ethics Committee of Wuhan Institute of Virology, CAS (Permit number: WIVA26201902).

### Statistical analysis

The Student’s t-test was used to determine if there were significant differences (*p* < 0.05) between different groups of infection. Kaplan-Meier survival curves were analysed by the log-rank test. The statistical analyses were performed using GraphPad Prism 8.0 software.

## Results

### Mapping the structures in SL and DB domains required for JEV replication in different host cells

**Figure 2.**
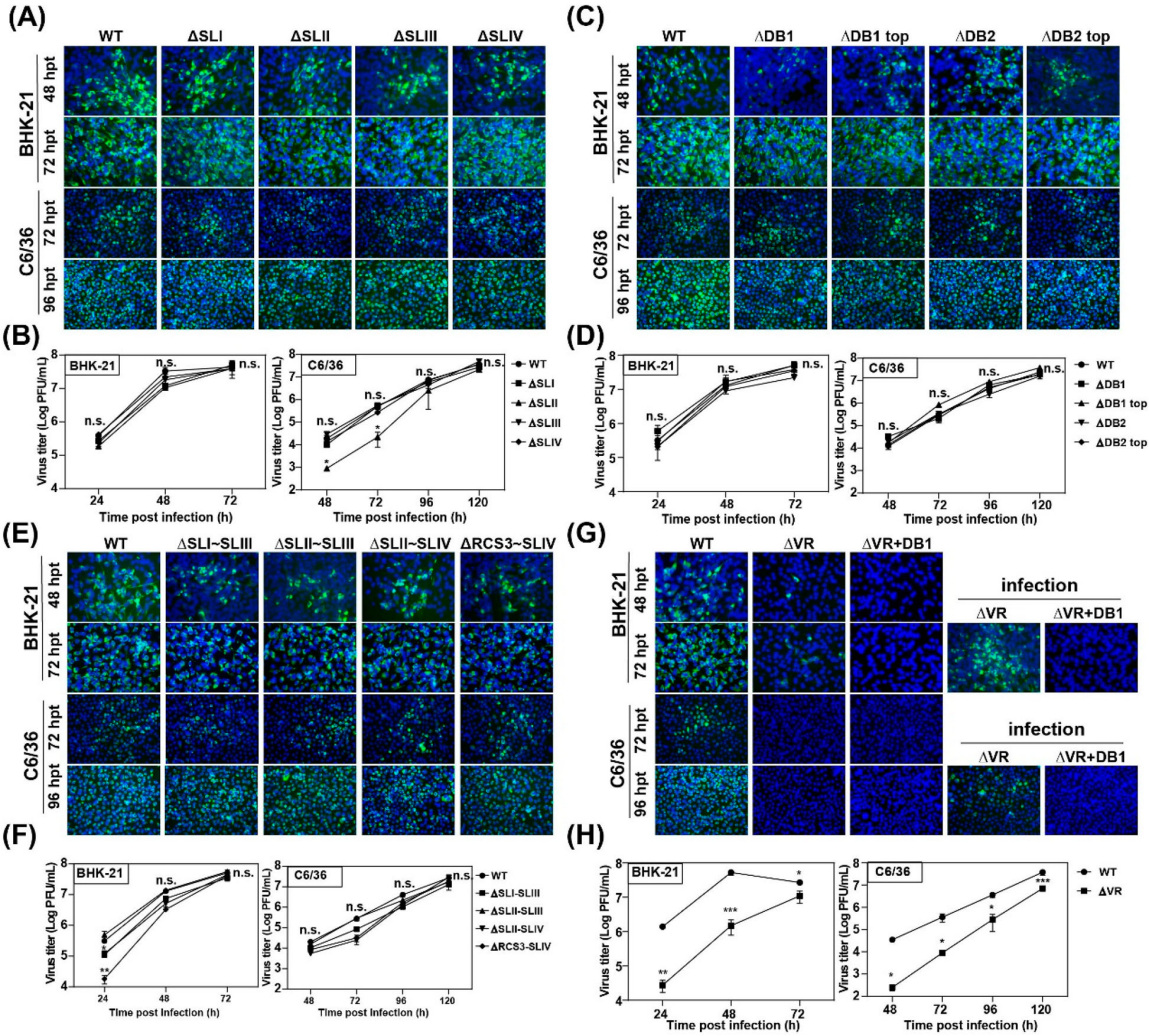
Detection of virus replication of different 3′ UTR deletion mutants in BHK-21 and C6/36 cells. (A) Equal amounts of wild-type (WT) or single structure deletion mutant RNAs in SL domain (ΔSLI, ΔSLII, ΔSLIII and ΔSLIV) were transfected into BHK-21 and C6/36 cells, respectively. Transfected cells were fixed at 48 and 72 h post transfection (hpt) in BHK-21 cells and at 72 and 96 hpt in C6/36 cells, respectively. The expression of E protein at the indicated time points was detected by IFA. The 4G2 monoclonal antibody and FITC-conjugated goat anti-mouse IgG were used as primary and secondary antibodies, respectively. (B) Growth kinetics of the WT and ΔSLI, ΔSLII, ΔSLIII, ΔSLIV mutant viruses in BHK-21 and C6/36 cells infected with the same MOI of 0.1. (C) IFA results of the transfected BHK-21 and C6/36 cells with WT, ΔDB1, ΔDB1 top, ΔDB2 or ΔDB2 top mutant RNAs, respectively. (D) Growth kinetics of WT, ΔDB1, ΔDB1 top, ΔDB2 and ΔDB2 top mutant viruses in BHK-21 and C6/36 cells infected with the same MOI of 0.1. (E) The expression of E protein was detected by IFA in the BHK-21 and C6/36 cells transfected with WT or combined structures deletion (ΔSLI∼SLIII, ΔSLII∼SLIII, ΔSLII∼SLIV and ΔRCS3∼SLIV) mutant RNAs, respectively. (F) Growth curves of WT and several structures deletion (ΔSLI∼SLIII, ΔSLII∼SLIII, ΔSLII∼SLIV, and ΔRCS3∼SLIV) mutant viruses in BHK-21 and C6/36 cells infected with the same MOI of 0.1. (G) The E protein expression of the WT, ΔVR and ΔVR + DB1 mutants in transfected and infected BHK-21 and C6/36 cells. After the mutant RNAs were transfected into BHK-21 and C6/36 cells, the cells were fixed at the indicated time points and subjected to IFA assay to determine the E protein expression and the supernatants were harvested and used to infect naïve BHK-21 and C6/36 cells, and the expression of E protein was detected at 72 and 96 hpi for BHK-21 and C6/36 cells, respectively. (H) Growth kinetics of WT and ΔVR mutant viruses in BHK-21 and C6/36 cells infected with an MOI of 0.1. n.s., indicates no statistical differences. *p < 0.05, **p < 0.01, *** *p* < 0.001.

Encouraged by the results that the host-specific replication of DENV2 was related to duplicated SL or DB secondary structures [7,8], we first analysed the effect of individual secondary structure deletion (Figure 1(B)) on JEV replication in mammalian and mosquito cells. The mutant RNAs were transfected into BHK-21 and C6/36 cells, respectively, and the expression of viral protein was analysed by IFA assay to characterize viral replication. As shown in Figure 2(A), in both BHK-21 and C6/36 cells, the percentage of IFA-positive cells produced by the mutants with individual structure deletion of SL domain (ΔSLI, ΔSLII, ΔSLIII and ΔSLIV) was similar to that of the WT virus. To further compare the viral replication efficiency, the growth kinetics of the mutant viruses in BHK-21 and C6/36 cells was determined by quantifying viral loads at different time points post infection at a multiplicity of infection (MOI) of 0.1. Consistent with the IFA results, the individual SL structure deletion mutants exhibited comparable growth kinetics as WT virus in both BHK-21 and C6/36 cells except that the ΔSLII mutant replicated slightly slower at the early stage of infection but reached high titres equal to that of WT virus at the late stage (Figure 2(B)). These results suggested that individual SL structure deletions didn’t affect virus replication in either BHK-21 or C6/36 cells. Similarly, the individual structure deletion mutants of domain DB, such as ΔDB1, ΔDB1 top, ΔDB2 and ΔDB2 top (Figure 1(B)), also had little influence on viral replication in either cell line (Figure 2(C and D)). These results demonstrated that any individual RNA structure deletion in domain SL and DB had no apparent effect on viral host-specific replication, which was inconsistent with the results of DENV2 [8,22,28].

To further analyse the functions of the duplicated structures in SL domain of JEV, we constructed various mutations with different combined SL structures deletion, including ΔSLI∼SLIII, ΔSLII∼SLIII, ΔSLII∼SLIV and ΔRCS3∼SLIV (Figure 1(B)), to retain only one or two SL structures in 3′ UTR. As shown by the IFA results (Figure 2(E)), the viral amplification of these deletion mutants was similar to that of WT virus in both BHK-21 and C6/36 cells at different time points. Growth curves of these mutants and WT viruses in BHK-21 and C6/36 cells were further analysed to compare their replication levels (Figure 2(F)). In BHK-21 cells, ΔSLI∼SLIII, ΔSLII∼SLIV and ΔRCS3∼SLIV deletion mutants replicated slightly slower at 24 h post infection (hpi), but reached comparable levels of viral titres as the level of WT virus at 48 and 72 hpi. In C6/36 cells, there was no significant difference in viral growth between these deletion mutants and WT virus. These results demonstrated that retaining one of the four SL structures in the SL domain was sufficient to maintain the replication of JEV in both mammalian and mosquito cells and the duplicated SL structures in 3′ UTR may not serve as a strategy for viral adaptation to mammalian or mosquito cells for JEV, which is different from the observation in DENV2 [8,22].

### Large fragment deletion significantly reduced JEV production

To further determine the RNA structures responsible for JEV genome replication, large fragment deletions, including the whole SL domain deletion (ΔVR) and the combined deletions of domain SL and DB (ΔVR + DB1), were constructed. The replication of these mutant viruses was also evaluated in both BHK-21 and C6/36 cells. As shown in Figure 2(G), the number of IFA-positive cells was dramatically decreased in ΔVR mutant RNA-transfected BHK-21 cells compared to that of WT, and no positive cells were observed in transfected C6/36 cells. To further evaluate the virus production of ΔVR in C6/36 cells, the supernatants harvested from ΔVR RNA-transfected BHK-21 cells were used to infect C6/36 cells, and small IFA-positive cell clusters were observed (Figure 2(G)), indicating that ΔVR also replicated poorly in mosquito cells. In addition, no IFA-positive cells were found after transfection of either BHK-21 or C6/36 cells with ΔVR + DB1 mutant RNA, indicating that this mutation was lethal for virus production. Growth curves of WT and ΔVR mutant viruses were compared in BHK-21 cells and C6/36 cells, and the results indicated that the replication of ΔVR mutant was significantly lower than that of WT virus at all time points post infection in both cell types, indicating that ΔVR mutation significantly reduced virus replication without host specificity (Figure 2(H)). These systematic deletion analyses demonstrated that the complete SL domain is indispensable for JEV virus replication in both mammalian and mosquito cells.

### Recovered viruses from the replication-deficient ΔVR mutant

**Figure 3.**
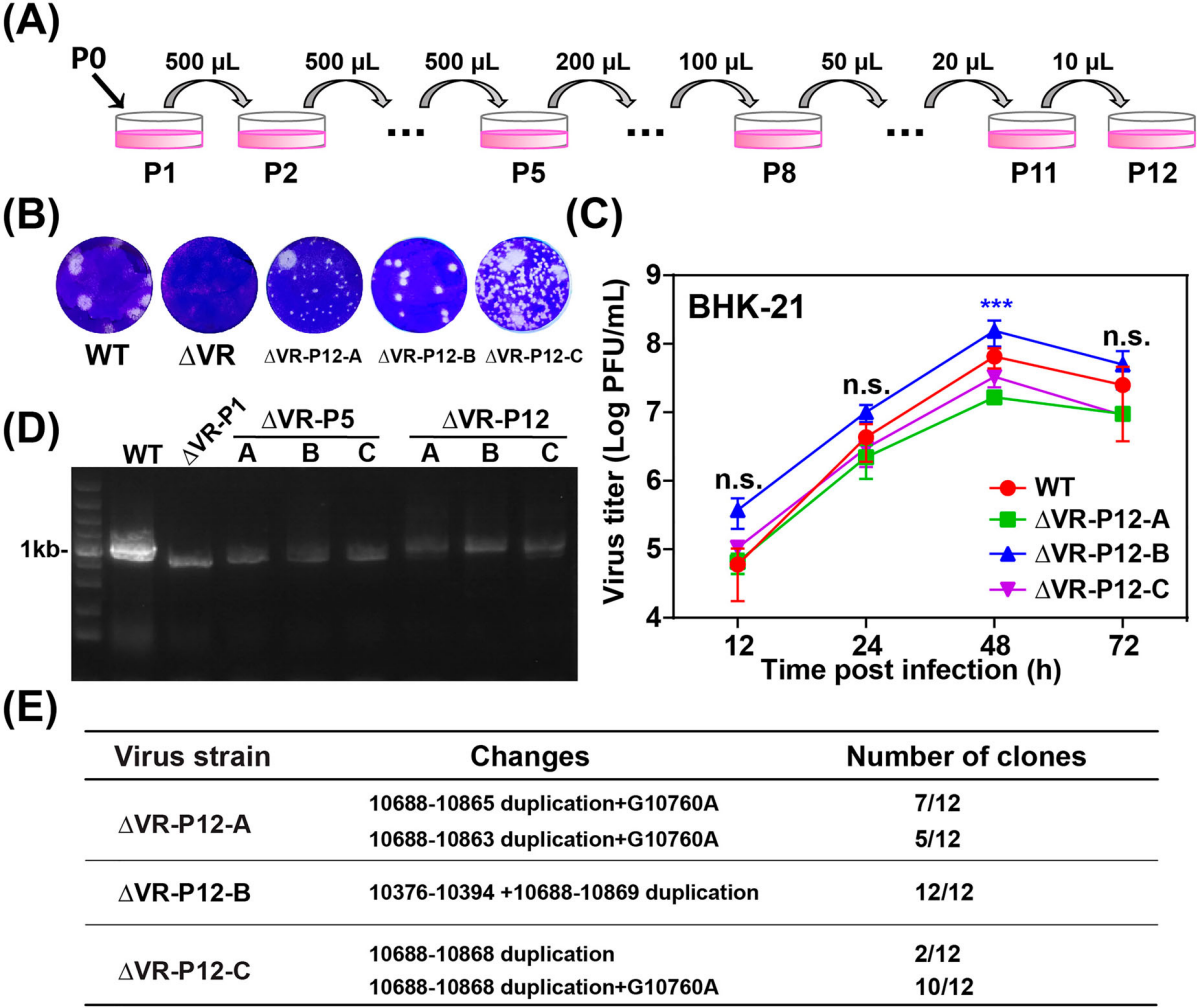
Characterization of ΔVR mutants passaged virus. (A) Schematic diagram of ΔVR mutant virus passaging in BHK-21 cells. (B) Plaque morphologies of the WT, ΔVR and ΔVR-P12-A/B/C viruses in BHK-21 cells. (C) Growth kinetics comparison between WT and ΔVR-P12-A/B/C viruses in BHK-21 cells. The WT and ΔVR-P12-A/B/C viruses infected BHK-21 cells at an MOI of 0.1, respectively, and the viral growth kinetics were determined by titration of the supernatants harvested at the indicated time points after infection by plaque assay. n.s., indicates no statistical differences. *** *p* < 0.001. (D) RT–PCR detection of the adaptation variation of ΔVR mutant during passaging. The primer pair, JEV-9841-F and JEV-10977-R (Table S1), was used to amplify the fragment spanning the terminal portion of NS5 and the proximal region of 3′ UTR. (E) The sequencing results of 12 single clones of ΔVR-P12-A/B/C viruses were obtained by plaque purification. The RT–PCR product spanning from NS5 to 3′ UTR of each purified virus was subjected to the sequencing analysis, and the whole-genome sequencing was performed for ΔVR-P12-B virus.

To study whether the replication deficiency of ΔVR mutant virus could be rescued by serial passages, three independent passages (A/B/C) were performed in BHK-21 cells in parallel. The supernatants collected from ΔVR mutant RNA-transfected BHK-21 cells were designated as P0 virus and subjected to continuous passaging in BHK-21 cells (Figure 3(A)). During the serial passage, no CPE was observed at the initial four passages, and mild CPE began to appear at P5. More obvious and faster CPE was observed from P6 to P12. The supernatants collected from P12 were designated as ΔVR-P12-A/B/C, respectively, and were subjected to growth kinetics and plaque morphology analyses in BHK-21 cells. ΔVR-P12-A/B/C viruses produced clear and visible plaques, which were different from the fuzzy plaques of ΔVR mutant (Figure 3(B)). In addition, the plaque morphologies of P12-A/C were not uniform in size, and P12-B formed uniform plaques with a smaller size than the WT virus. By comparing the growth kinetics in BHK-21 cells, it was found that ΔVR-P12-A/C replicated similarly to WT virus in BHK-21 cells, and ΔVR-P12-B showed efficient replication with even higher peak titres than WT virus (Figure 3(C)), confirming that virus replication defect of ΔVR mutant was recovered after serial passage.

**Figure 4.**
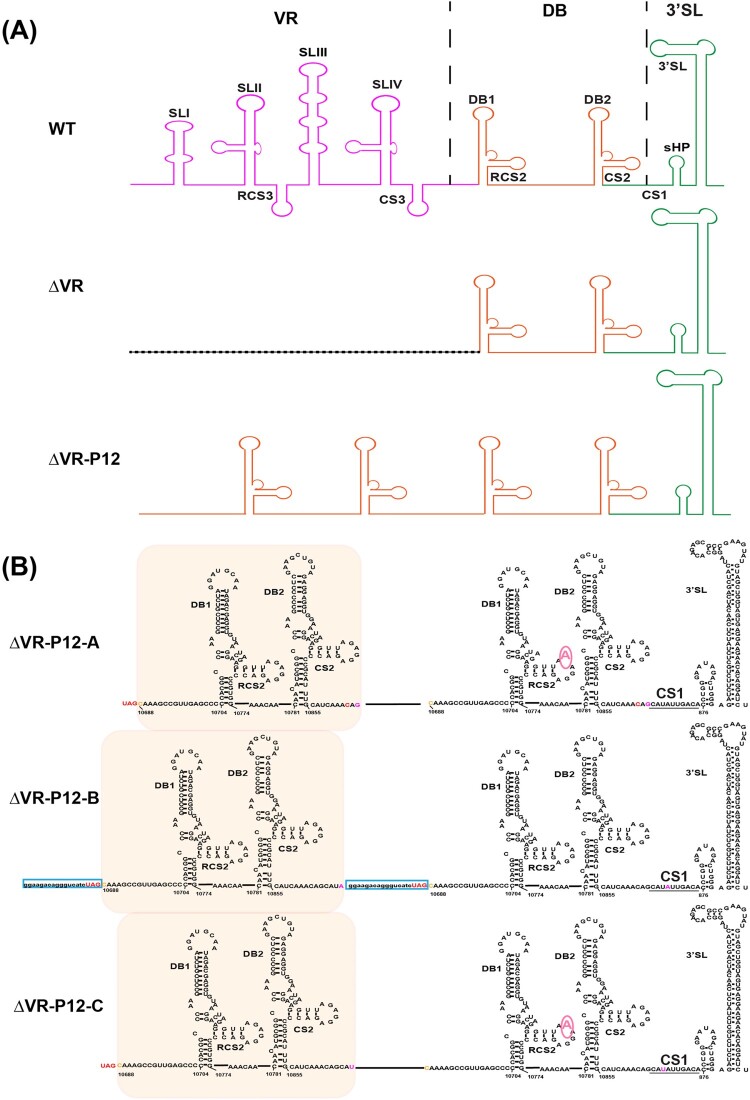
Diagram of 3′ UTR structure and sequences of passaged ΔVR-P12-A/B/C viruses. (A) Schematic of the 3′ UTR structure of JEV WT, ΔVR and ΔVR-P12 viruses. (B) Detailed 3′ UTR sequences of the representative clone of ΔVR-P12-A/B/C viruses were presented. There was a G10760A site mutation in the second DB1 structure in ΔVR-P12-A/C viruses, and there was an additional 19 nucleotides duplication within the C-terminus of NS5 in ΔVR-P12-B virus.

To identify the revertant mutations in the P12 viruses, viral RNAs were extracted for RT–PCR using primers spanning the region from NS5 to 3′ UTR. WT and ΔVR P1 viral RNAs were used as controls. As expected, the WT and ΔVR P1 viruses produced 1.1 kb and 843 bp fragments (Figure 3(D)), respectively. In contrast, ΔVR-P5-A/B/C and P12-A/B/C generated about 900 bp and 1 kb products (Figure 3(D)), respectively, suggesting the sequences insertion mutation in their genome. To identify the revertant mutations, 12 plaque purified clones of the ΔVR-P12-A/B/C viruses were subjected to sequencing the NS5 to 3′ UTR region. The data showed that the additional DB1+DB2 sequences with minor differences were identified besides the original DB1+DB2 sequences in ΔVR-P12-A/B/C viruses (Figure 3(E)), producing the DB domain duplications in 3′ UTR (Figure 4). It was also found that there was a G10760A nucleotide mutation within the second duplicated DB1 structure in A and C strains, and a 19 nucleotides duplication within the C-terminus of NS5 sequence (10376–10394) in B strain, respectively (Figures 3(E) and 4). The ΔVR-P12-B was further sequenced for the entire genome, and a point amino acid mutation D389G in E protein was identified. The D389G mutation frequently emerged during the WT or other mutant JEV viruses passaging in BHK-21 cells in our laboratory, suggesting that it was a cell-adapted mutation of JEV SA14 in BHK-21 cells. We next chose the adaptive mutations in the 3′ terminal region of ΔVR-P12-B to validate the role of DB duplication in restoring virus replication.

### Complete DB duplication recovered the replication capability

**Figure 5.**
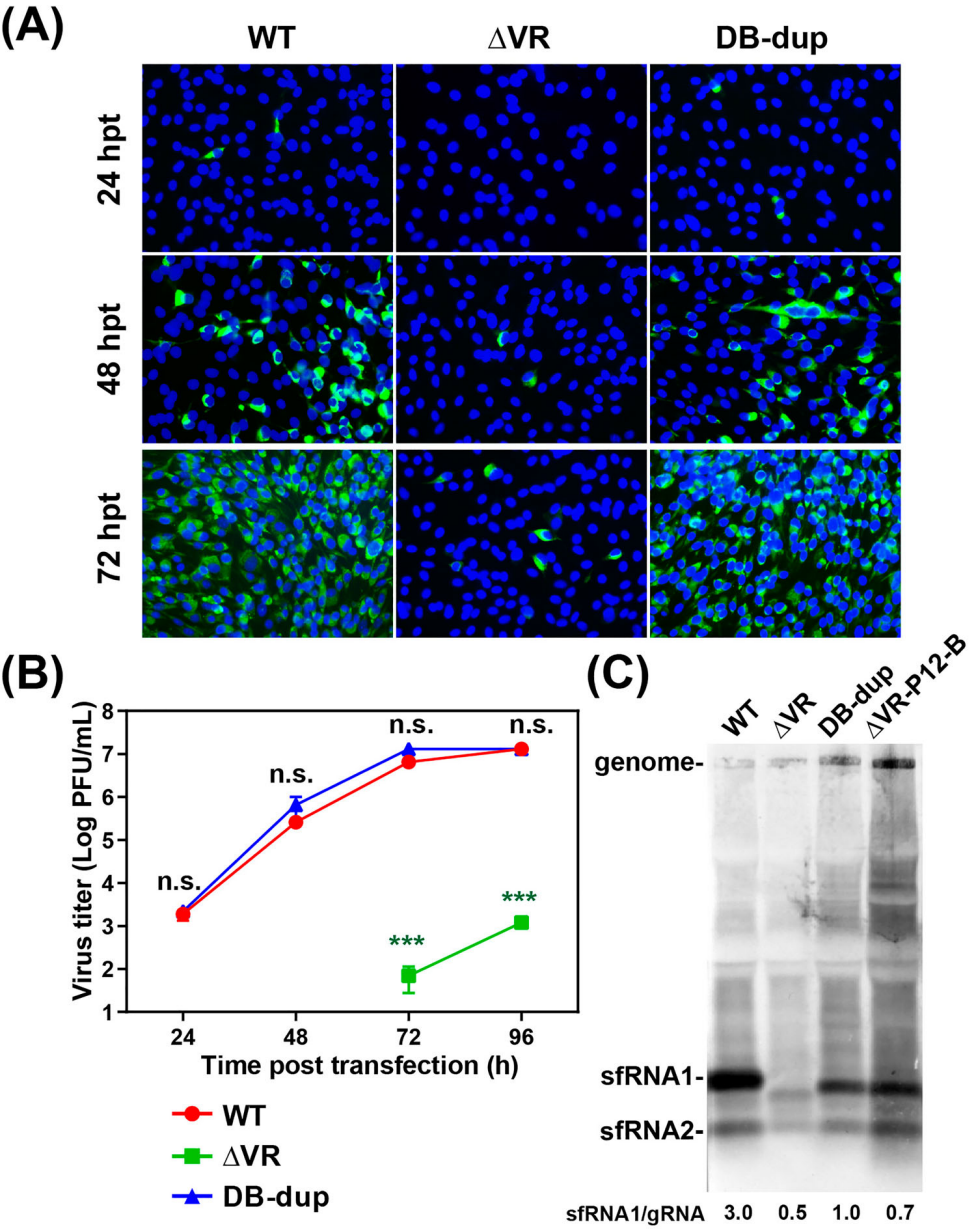
Characterization of the adaptive mutations of ΔVR revertant viruses on virus replication and sfRNA production. (A) The expression of E protein in the mutant RNAs-transfected BHK-21 cells. Equal amounts of the mutant RNAs derived from the full-length infectious clones were transfected into BHK-21 cells, and IFA was performed at the indicated time points. (B) Growth kinetics of ΔVR, DB-dup and WT viruses in BHK-21 cells. The supernatants from RNA-transfected cells at different time points were collected and subjected to plaque assay to determine the virus titres. n.s., indicates no statistical significance. *** *p* < 0.001. (C) Northern blotting analysis of the total RNAs extracted from BHK-21 cells infected with WT, DB-dup, and ΔVR-P12-B viruses, respectively. Bands intensities of sfRNAs and gRNA were quantified using Image J software, and the ratios of sfRNA1/gRNA were shown below.

To determine the effect of the DB duplication (DB-dup) mutation on virus replication, we constructed the full-length infectious clone including DB-dup mutation using the ΔVR mutant as the backbone. The mutant RNAs were transfected into BHK-21 cells, and at different time points, the cells were fixed for IFA and the supernatants were collected to determine the viral production. As shown in Figure 5(A), in contrast to only small amounts of positive cells observed in cells transfected with ΔVR RNA, approximately 100% positive cells were observed in cells transfected with WT or DB-dup RNA at 72 hpt, indicating that the replication defect of ΔVR was rescued by DB-dup mutation. By comparing the growth kinetics of P0 viruses, we confirmed that the DB-dup could rescue the replication of ΔVR mutant to a similar level of WT virus (Figure 5(B)). These results indicated the important function of DB-dup in recovering virus replication.

### Complete DB duplication produced sfRNAs patterns similar to WT virus

The sfRNAs of flaviviruses have been demonstrated to be critical for viral cytopathic effect in cell culture and pathogenicity in mice [11]. The sfRNAs are generated from the degradation of genomic RNA by cellular exoribonuclease Xrn1 which is stalled by SL or DB structures in 3′ UTR to produce different lengths of sfRNAs [10,11,23–25]. WNV, DENV and YFV mutants with the defect of sfRNA production replicated poorly in cell culture [19,24,25]. To identify whether the replication defect of ΔVR was related to the loss of sfRNAs, we detected the sfRNAs production of ΔVR and DB-dup mutants. Total RNAs were extracted from the different viruses infected cells for the northern blotting assay. As shown in Figure 5(C), ΔVR generated the sfRNAs pattern of distinct from that of WT. Specifically, abundant sfRNA1 was observed in WT-infected BHK-21 cells, which is in agreement with previous studies [29,30], whereas smaller-sized and less amount of sfRNA1 was accumulated in ΔVR-infected cells. Interestingly, the patterns of sfRNAs produced by DB-dup and ΔVR-P12-B were similar to that of WT, except that the size of sfRNA1 was smaller than WT virus, which was consistent with the 3′ UTR sequence length of DB-dup and WT virus (Figure 5(C)). In addition, a weak band of sfRNA2 was observed, and no sfRNA3 or sfRNA4 was found in WT-, DB-dup- and ΔVR-P12-B- infected cells, which is consistent with the finding in DENV2, but different from that in WNV [10,31]. By calculating the ratio of sfRNA1 to gRNA (sfRNA:gRNA), we found that the DB-dup and ΔVR-P12-B viruses only generated partially increased sfRNA:gRNA ratio compared to ΔVR mutant, which was much lower than that of WT virus (Figure 5(C), lower panel). These results demonstrated that the ΔVR mutant affected the pattern and amount of sfRNAs production during virus infection, and DB-dup could partially recover these defects.

### Complete DB duplication in 3′ UTR attenuated viral virulence in C57BL/6 mice

**Figure 6.**
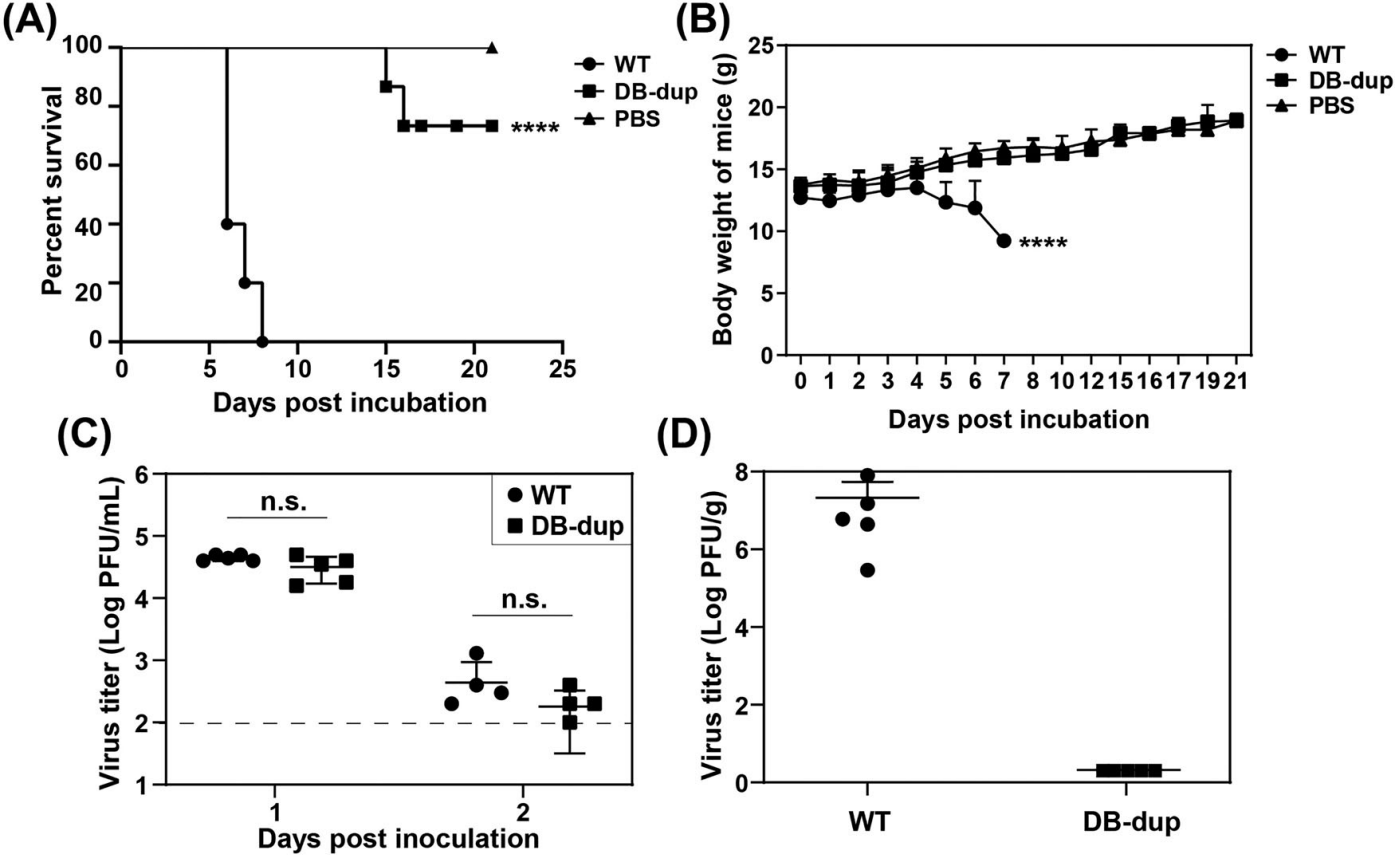
DB-dup mutation attenuated viral virulence in C57BL/6 mice. (A) Survival analysis of mice infected with WT and DB-dup viruses. Three-week-old C57BL/6 mice were i.p. inoculated with 1×10^7^ PFU of JEV WT and DB-dup virus, respectively, and then monitored for clinical symptoms and mortality over 21 days. The average survival rate of the infected mice from three independent assays was presented. **** *p* < 0.0001. (B) Body- weight changes of mice infected with JEV were monitored for 21 days. **** *p* < 0.0001. (C) Viremia on the first and the second days post infection by plaque assay in BHK-21 cells. The dotted line indicated the detection limit (100 PFU/mL). n.s., indicates no significant difference. (D) Virus loads in the brains of mice infected with WT virus at 6 dpi or in survived mice infected with DB-dup virus at 21 dpi.

The structure of flavivirus 3’ UTR and the production of sfRNAs have been reported as one of important determinants of viral pathogenicity [9,11]. Our recent study identified that a WNV mutant carrying a replacement of SL and DB domains of 3′ UTR with internal poly(A) tract was highly attenuate [32]. Although DB-dup virus contained duplicated DB sequences, there was still a large fragment of SL domain missing from its genome. To determine the effect of DB-dup mutation on viral virulence, 1×10^7^ PFU WT or DB-dup viruses were intraperitoneally (i.p.) injected into three-week-old C57BL/6 mice which are highly sensitive to JEV infection [33]. The body-weight change and mortality of the infected mice were monitored for 21 days. Mice infected with WT virus started to develop severe symptoms including ruffled fur, limb paralysis and weight loss at 5 days post injection (dpi) (Figure 6(B)), and all mice died at 8 dpi (Figure 6(A)). In contrast, the DB-dup virus-infected mice developed symptoms after 7 dpi with an average mortality rate of 26% throughout the observation period (Figure 6(A)). Body-weight changes of the survived mice inoculated with DB-dup were comparable to those of the PBS-infected control mice (Figure 6(B)), suggesting that there were no signs of illness in the survived mice. Moreover, viremia in mice infected with WT or DB-dup mutant viruses was detected on day 1 and day 2 after infection, and the results showed that there was no significant difference between the two groups (Figure 6(C)). The average viral loads in the brains reached approximately 10^7^ PFU/g in WT-virus-infected mice at 6 dpi, whereas the virus titre was undetectable in DB-dup infected group even at 21 dpi (Figure 6(D)). We speculated that the attenuation of DB-dup virus may be due to the reduced ability to cross the blood–brain barrier or the rapid clearance once the virus reaches the brain. These results demonstrated that although the DB-dup mutant exhibited similar replication efficiency to WT in cell culture, the virulence of DB-dup was attenuated in C57BL/6 mice compared with the WT virus.

## Discussion

Many studies have shown that the secondary RNA structures in flavivirus 3′ UTR are important elements in viral replication, host-adaptation and pathogenicity [5,10,11,25]. Among these structures, sHP-SL domain is indispensable for genome cyclization and RNA synthesis [5,6,34], and the SL and DB domain play essential roles in virus replication, especially on the host-specific replication of DENV virus in mammalian and mosquito cells [22,28]. In the present study, we found that the duplicated structures of SL and DB are not host-specific for JEV replication (Figure 2(A–F)), suggesting different evolutionary strategies for host-specific fitness among flaviviruses. Moreover, the entire SL domain sequence deletion (ΔVR) dramatically reduced virus replication, and the ΔVR + DB1 mutant containing the entire SL domain and DB1 sequences deletion was lethal for virus replication (Figure 2(G and H)), indicating the essential function of the complete SL domain during JEV replication in both mammalian and mosquito cells. This result was consistent with a Δ307 mutant (retaining 30 nucleotide acids of 5′ proximal in 3′ UTR of JEV strain RP9, similar to ΔVR in this study) which abolished viral replication [35], but was different from a previous study on another JEV CNU/LP2 strain, in which MUTΔ1-284 (similar to ΔVR) showed similar replication level to WT, and MUTΔ1-381 (similar to ΔVR + DB1) replicated moderately slower than WT [15]. The contradictory results suggested that the varied functions of 3′ UTR in viral replication of different virus strains, which may be caused by sequence differences in the 3′ UTR.

To further identify the function of the SL domain during replication, we performed continuous passaging of the ΔVR mutant to recover the viral replication efficiency (Figure 3). Besides the original DB1+DB2 sequences, additional DB1+DB2 sequences were identified to recover replication of ΔVR mutant (Figures 4 and 5). In a previous study of DENV2, the replication deficiency of DB1+DB2 sequences deletion mutant (M8) could be restored by the VR or SL sequences duplication [31]. These results suggested that the duplicated structures may evolve for flavivirus replication besides host-specific fitness.

The production of sfRNAs is a common feature for flavivirus [11]. In our study, we found that ΔVR mutation produced smaller and fewer sfRNAs than WT virus, while the passaged virus ΔVR-P12-B and the rescued DB-dup mutant virus partially recovered the formation of sfRNAs and produced similar sfRNAs patterns to WT virus (Figure 5(C)), indicating the partial consistency between virus replication level and sfRNA production. Generally, the duplicated SL (SLII and SLIV) and DB (DB1 and DB2) structures are required for the generation of different lengths of sfRNA1, sfRNA2, sfRNA3 and sfRNA4, respectively [11,25]. Both sfRNA1 and sfRNA2 were detected for JEV SA14 strain (Figure 5(C)), and only sfRNA1 was detected in the previous study for the JEV RP9 strain [30]. It is likely that the different sequences of virus strains may influence the sfRNAs production patterns of JEV, just like the findings in DENV2 [36].

There has been a growing body of evidence showing that sfRNAs formation is closely related to viral pathogenicity [11,25]. The mutant flavivirus with defect of sfRNA1 production attenuated viral virulence in mice [10,11]. Our results showed that although DB-dup mutant produced similar sfRNA patterns to WT, the ratio sfRNA:gRNA of DB-dup virus was still lower than that of WT. In addition, DB-dup mutant virus attenuated viral virulence in mice (Figure 6), indicating that the decreased sfRNA:gRNA ratio may be one of the factors leading to the pathogenicity attenuation for DB-dup virus [37]. Furthermore, it has been reported that the specific sfRNA sequence of DENV2 could interact with cellular TRIM25 protein [37] or the novel interferon (IFN) response regulator G3BP1, G3BP2 and CAPRIN1 proteins [38], further affecting IFN expression of the infected cells. Other host proteins, such as La, DDX5 or p100, are also reported to interact with flavivirus 3′ UTR to influence virus replication [39–41]. Moreover, researchers found that the 3′ UTR of the low-virulent strain of TBEV could specifically interact with the host proteins CSDE1, FMRP, ILF3 and STRBP [42]. These results indicate that the interactions between flavivirus 3′ UTR and host proteins are involved in viral replication and pathogenicity. We speculate that the entire SL domain deletion in the DB-dup virus may also disrupt the interaction between 3′ UTR and host factors that are essential for virus pathogenicity, which needs to be further explored in future studies.

## Supplementary Material

Supplemental Material
