# Supplementary material for: Sequence duplication in 3′ UTR modulates virus replication and virulence of Japanese encephalitis virus
Source: Emerg Microbes Infect. 2021 Dec 21;11(1):123–35. doi: 10.1080/22221751.2021.2016354 (PMC8725919; doi:10.1080/22221751.2021.2016354)
Supplement: Supplemental Material [file TEMI_A_2016354_SM5467.docx]

**Supplementary Materials for**

**Sequence Duplication in 3ʹ UTR Modulates Virus Replication and Virulence of Japanese encephalitis virus**

Qiu-Yan Zhang^1, 2^, Si-Qing Liu^3^, Xiao-Dan Li^4^, Jia-Qi Li^3,5^, Ya-Nan Zhang^3^, Cheng-Lin Deng^3^, Hong-Lei Zhang^6^, Xu-Fang Li^1^, Chun-Xiao Fang^1^, Feng-Xia Yang^1^, Bo Zhang^1, 2, 3,*^, Yi Xu^1, 2,*^, Han-Qing Ye^3,*^

*Corresponding author: Han-Qing Ye ([yehq@wh.iov.cn](mailto:yehq@wh.iov.cn)), Yi Xu ([xuyi70@163.com](mailto:xuyi70@163.com)) or Bo Zhang [(zhangbo@wh.iov.cn)](mailto:(zhangbo@wh.iov.cn))

This file contains:

Figure S1 and Table S1

**Figure S1. The sketches of 3ʹ UTR secondary structures of different flaviviruses.**


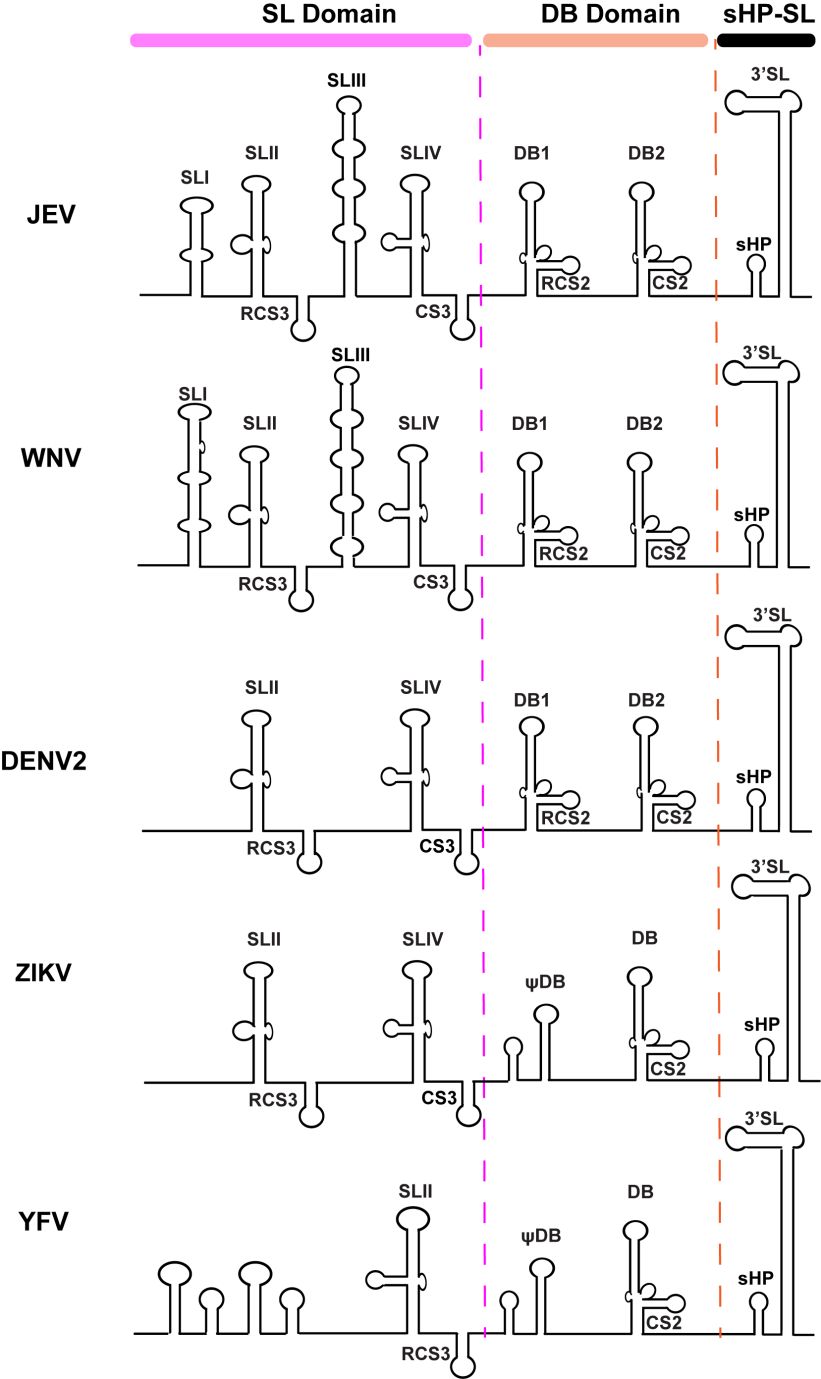


**Figure S1. The sketches of JEV, WNV, DENV2, ZIKV and YFV** **3ʹ UTR secondary structures.** Based on the differences of sequences conservation, 3ʹ UTR is divided into three domains: SL domain, DB domain and sHP-SL domain, and labeled with purple, orange or black thick lines, respectively.

**Table S1. Details of primers used in the infectious clones construction, RT-PCR amplification and DNA probe for northern blotting analysis.**

| **Primer Name** | **Genome position** | **Utilities** | **Sequence** |
| --- | --- | --- | --- |
| JEV-8881-NS5-F | 8851 | Fusion PCR | TAAAGAAAGTCAATAGCAACG |
| PACYC-R | pACYC vector | Fusion PCR | CTGCCACATGAAGCACTTC |
| ΔSLI-F | 10395-10421 | ΔSLI mutant construction | CAGGAAGACAGGGTCATCTGTAAATAATGTAAATGAG |
| ΔSLI-R | 10395-10421 | ΔSLI mutant construction | CTCATTTACATTATTTACAGATGACCCTGTCTTCCTG |
| ΔSLII-F | 10457-10514 | ΔSLII mutant construction | AGAAAATGCATGCATATGAGTCCCAGGAGGACTGGGTTAACAAATCTGACAACAGAA |
| ΔSLII-R | 10457-10514 | ΔSLII mutant construction | TTCTGTTGTCAGATTTGTTAACCCAGTCCTCCTGGGACTCATATGCATGCATTTTCTCA |
| ΔSLIII-F | 10541-10613 | ΔSLIII mutant construction | GAGGACTGGGTTAACAAACCAACGTCAGGCCACAAATT |
| ΔSLIII-R | 10541-10613 | ΔSLIII mutant construction | AATTTGTGGCCTGACGTTGGTTTGTTAACCCAGTCCTC |
| ΔSLIV-F | 10618-10884 | ΔSLIV mutant construction | TGGAAGTTGAAAGACCAACAGCCCCAGGAGGACTGG |
| ΔSLIV-R | 10618-10884 | ΔSLIV mutant construction | CCAGTCCTCCTGGGGCTGTTGGTCTTTCAACTTCCA |
| ΔDB1-F | 10706-10772 | ΔDB1 mutant construction | CAAAGCCGTTGAGCCCCCGGAAACAACAACATGCGG |
| ΔDB1-R | 10706-10772 | ΔDB1 mutant construction | CCGCATGTTGTTGTTTCCGGGGGCTCAACGGCTTTG |
| ΔDB1 top-F | 10716-10741 | ΔDB1 top mutant construction | GAGCCCCCACGGCCCAAGTGTAAGGACTAGAGGTTAGAGGAGACCCCGTGGAAACAAC |
| ΔDB1 top-R | 10716-10741 | ΔDB1 top mutant construction | GTTGTTTCCACGGGGTCTCCTCTAACCTCTAGTCCTTACACTTGGGCCGTGGGGGCTC |
| ΔDB2-F | 10781-10885 | ΔDB2 mutant construction | GAGACCCCGTGGAAACAACATCAAACAGCATATTGA |
| ΔDB2-R | 10781-10885 | ΔDB2 mutant construction | TCAATATGCTGTTTGATGTTGTTTCCACGGGGTCTC |
| ΔDB2 top-F | 10797-10819 | ΔDB2 top mutant construction | AACAACATGCGGCCCAAGTGGAAGGACTAGAGGTTAGAGGAGACCCCGCATTTGCATCA |
| ΔDB2 top-R | 10797-10819 | ΔDB2 top mutant construction | TGATGCAAATGCGGGGTCTCCTCTAACCTCTAGTCCTTCCACTTGGGCCGCATGTTGTT |
| ΔSLI~SLIII-F | 10395-10613 | ΔSLI~SLIII mutant construction | CAGGAAGACAGGGTCATCTAGCCAACGTCAGGCCACAAATT |
| ΔSLI~SLIII-R | 10395-10613 | ΔSLI~SLIII mutant construction | AATTTGTGGCCTGACGTTGGCTAGATGACCCTGTCTTCCTG |
| ΔSLII~SLIII-F | 10457-10613 | ΔSLII~SLIII mutant construction | AGAAAATGCATGCATATGCCAACGTCAGGCCACAAATT |
| ΔSLII~SLIII-R | 10457-10613 | ΔSLII~SLIII mutant construction | AATTTGTGGCCTGACGTTGGCATATGCATGCATTTTCT |
| ΔSLII~SLIV-F | 10457-10564 | ΔSLII~SLIV mutant construction | AGAAAATGCATGCATATGCAGCCCCAGGAGGACTGGGTTACCAAAGCCGTTG |
| ΔSLII~SLIV-R | 10457-10564 | ΔSLII~SLIV mutant construction | CTTTGGTAACCCAGTCCTCCTGGGGCTGCATATGCATGCATTTTCTCATTTAC |
| ΔRCS3~SLIV-F | 10518-10664 | ΔRCS3~SLIV mutant construction | GTGCTGCCTGCGTCTCAGTCAGCCCCAGGAGGACTGGGTTACCAAAGCCGTTGAGCCCC |
| ΔRCS3~SLIV-R | 10518-10664 | ΔRCS3~SLIV mutant construction | GGGGCTCAACGGCTTTGGTAACCCAGTCCTCCTGGGGCTGACTGAGACGCAGGCAGCAC |
| ΔVR-F | 10395-10687 | ΔVR mutant construction | CAGGAAGACAGGGTCATCTAGCAAAGCCGTTGAGCCCCCAC |
| ΔVR-R | 10395-10687 | ΔVR mutant construction | GTGGGGGCTCAACGGCTTTGCTAGATGACCCTGTCTTCCTG |
| ΔVR+DB1-F | 10395-10804 | ΔVR+DB1 mutant construction | AGGAAGACAGGGTCATCTAGCATGCGGCCCAAGCCCCCTCG |
| ΔVR+DB1-R | 10395-10804 | ΔVR+DB1 mutant construction | CGAGGGGGCTTGGGCCGCATGCTAGATGACCCTGTCTTCCT |
| JEV-3’UTR-HDVr-F | pACYC vector | DB-dup mutant construction | AACACAGGATCTGGGTCGGCATGGCATCTCC |
| JEV-3’UTR-HDVr-R | pACYC vector | DB-dup mutant construction | GCCATGCCGACCCAGATCCTGTGTTCTTCCTC |
| JEV-9841-F | 9841 | RT-PCR | TGATGAAAGATGGAAGGAGTA |
| JEV-10847-F | 10847 | PCR for DNA probe | CCGCATTTGCATCAAACAGC |
| JEV-10977-R | 10977 | RT-PCR / PCR for DNA probe | AGATCCTGTGTTCTTACTCAC |
